# Behavioural profiling of autism connectivity abnormalities

**DOI:** 10.1192/bjo.2019.102

**Published:** 2020-01-22

**Authors:** William Snyder, Vanessa Troiani

**Affiliations:** Student, Program in Neuroscience, Bucknell University; Research Assistant, Autism and Developmental Medicine Institute, Geisinger, USA; Assistant Professor, Autism and Developmental Medicine Institute, Geisinger; and Department of Basic Sciences, Geisinger Commonwealth School of Medicine, USA

**Keywords:** Autism spectrum disorder, neuroimaging, fMRI, meta-analysis

## Abstract

**Background:**

Brain regions are functionally diverse, and a given region may engage in a variety of tasks. This functional diversity of brain regions may be one factor that has prevented the finding of consistent biomarkers for brain disorders such as autism spectrum disorder (ASD). Thus, methods to characterise brain regions would help to determine how functional abnormalities contribute to affected behaviours.

**Aims:**

As the first illustration of the meta-analytic behavioural profiling procedure, we evaluated how the regions with disrupted connectivity in ASD contributed to various behaviours.

**Method:**

Connectivity abnormalities were determined from a published degree centrality group comparison based on functional magnetic resonance imaging data from the Autism Brain Imaging Data Exchange. Using BrainMap's database of task-based neuroimaging studies, behavioural profiles were created for abnormally connected regions by relating these regions to tasks activating them.

**Results:**

Hyperconnectivity in ASD brains was significantly related to memory, attention, reasoning, social, execution and speech behaviours. Hypoconnectivity was related to vision, execution and speech behaviours.

**Conclusions:**

The procedure outlines the first clinical neuroimaging application of a behavioural profiling method that estimates the functional diversity of brain regions, allowing for the relation of abnormal functional connectivity to diagnostic criteria. Behavioural profiling and the computational insights it provides can facilitate better understanding of the functional manifestations of various disorders, including ASD.

## Overview

Clinical neuroimaging seeks to find consistent differences between control and patient brains in order to describe neural mechanisms for behavioural differences between groups. Autism neuroimaging has used structural and functional magnetic resonance imaging (MRI) methods to describe neurological differences that contribute to the autism spectrum disorder (ASD) behavioural phenotype. The ASD behavioural phenotype is heterogeneous,^1^ but all people with ASD share the same core symptoms evident in behaviour. Thus, a method that allowed one to evaluate the relationship between connectivity differences and core behavioural differences would be useful. Functional connectivity, a method measuring functional synchrony across brain regions, has been used to characterise the degree of abnormal brain connectivity present in individuals with ASD. The results of such brain connectivity analyses are often related to behaviour by acknowledging abnormalities in regions that have been previously implicated in behaviours involved in the ASD phenotype. For example, ‘social’ brain networks contribute to social deficits in ASD (or any other brain network/behaviour combination). Although these evaluations are not inaccurate, there is increasing realisation that certain brain regions are involved in a wide variety of behaviours and cognitive tasks.^2,3^ Thus, statistical measures that quantify the degree of involvement of specific brain regions in behaviours affected by a disorder would be valuable.

## Meta-analysis in brain mapping

Anderson and colleagues^4^ developed a meta-analytic approach to quantify each brain region's degree of involvement in various behaviours, but the method has not yet been applied in a clinical context. Anderson described brain regions that participate in many tasks as ‘functionally diverse’ and characterised brain regions' tendencies to participate in certain tasks as ‘functional biases’, with the majority of brain regions demonstrating some degree of functional diversity. This method, which Anderson and colleagues termed ‘functional fingerprinting’, allows one to quantify a given brain region's functional biases. (Please note that this type of ‘functional fingerprinting’ meta-analysis is unrelated to the functional fingerprinting of Finn *et al*^5^; to avoid confusion, we have adopted the term ‘behavioural profiling’ for the work described here.) Using the methods described by Anderson *et al*,^4^ one can plot a brain region's behavioural profile as the proportions of functional bias towards a set of behavioural domains, giving insight to the involvement of brain regions across a variety of behavioural domains.

Meta-analytic databases dominantly include data on healthy participants, which can be useful for describing neural computations affected in a disorder. Task-based meta-analyses using the Neurosynth (http://neurosynth.org) and BrainMap (http://brainmap.org) databases have made it possible to pool data from many experiments to describe networks and brain regions, enabling computation of additional metrics that would be unavailable for single experiments (such as with functional diversity). Meta-data relate tasks and foci activated under task conditions compared with control conditions, and these task-based meta-data form the basis for functional MRI (fMRI) meta-analyses. Functional diversity reflects how brain regions are reused for various tasks, and the neural reuse described by Anderson^2^ may also reflect a reuse of neural computations. Meta-analysis of healthy participant data can reveal the functional or task biases of a brain region's underlying computation. Characterising brain regions affected by a disorder would then reveal the functional biases of healthy computations that the condition disrupts.

## Application of behavioural profiling

Here, we apply a novel behavioural profiling method to establish whether group differences in resting state functional connectivity MRI (rs-fcMRI) between individuals with ASD and controls exist in regions functionally biased towards behaviours disrupted in ASD. We reasoned that if affected regions in the brains of people with ASD were related to behaviours affected by ASD (e.g. ‘social’ behaviours), it would be expected that foci landing within the affected regions would tend to have meta-data aligning with those behaviours (e.g. foci from ‘social’ tasks). In the present study, we use meta-data derived from task-based meta-analyses to quantify functional biases of atypically connected brain regions in an ASD cohort. The data-driven behavioural profiling method, adapted from the functional fingerprinting method of Anderson *et al*^4^, used BrainMap meta-data to quantify functional biases of brain regions. We expected that brain regions that showed atypical connectivity in ASD would exhibit functional biases towards core ASD-affected behaviours. The following study proposes behavioural profiling as a metric for quantifying clinical neuroimaging results to give insight into the neural underpinnings of core symptoms.

## Method

### Overview of behavioural profiling

**Fig. 1:**
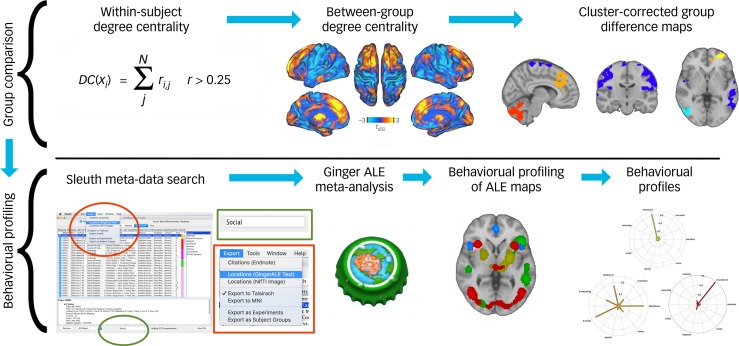
Behavioural profiling pipeline using degree-centrality difference maps as ROIs.

To reveal the functional biases of functionally connectivity abnormality regions of interest (ROIs) in ASD, a data-driven behavioural profiling approach, adapted from Anderson's ‘functional fingerprinting’ meta-analysis,^4^ was applied using BrainMap data. The procedure (outlined in Fig. 1) involved determining ROIs related to the disorder and quantifying each ROI's functional bias towards a set of behavioural domains with descriptive power for brain function.

Degree centrality group difference maps of hyperconnectivity and hypoconnectivity served as ROIs for behavioural profiling, generating one radially plotted behavioural profile per group difference cluster. ROIs were input to the Sleuth application (first image in the Behavioural Profiling row)^6^ and meta-data were filtered with Sleuth's filter tool created at our request (available in v3.0a2 and above). The domain ‘social’ is shown as an example of a behavioural domain with which the workspace can be filtered (highlighted in green). Domain-specific workspaces were exported (highlighted in red) for GingerALE meta-analysis, and activation likelihood maps determined the degree of functional bias towards behavioural domains within the ROI's behavioural profile. The degree centrality equation was adapted from Zuo *et al*^7^ and the description from Holiga *et al.*^8^ ALE, activation likelihood estimation; ROI, region of interest.

### Determining ROIs: cohort and functional connectivity differences

The focus of the current paper was not to identify novel regions associated with connectivity differences in ASD. ROIs can be determined based on connectivity differences that are determined from new or existing results using a variety of resting-state connectivity analysis methods. To emphasise the behavioural profiling method and not ASD connectivity differences *per se*, we chose to implement this behavioural profiling method on an established data-set of reproducible resting-state functional connectivity alterations.^8^ The cohort used in determining functional connectivity differences included 299 ASD and 376 typically developed participants from the first cohort for the multi-site Autism Brain Imaging Data Exchange (ABIDE I).^9^ Participants included both male and female individuals with IQ > 70, and were sampled from child, adolescent and adult age ranges. ASD subjects included all ASD diagnoses in the ABIDE database (autism, Asperger syndrome, or pervasive developmental disorder not otherwise specified). Detailed subject demographics can be found in Holiga *et al*.^8^ Voxel-wise degree centrality maps of rs-fcMRI were computed by Holiga *et al*^8^ to compare functional connectivity between the ASD and typically developed cohorts. Degree centrality is a functional connectivity-based metric that is computed for each voxel as the sum of the correlation coefficients (*r*) of that voxel's time series with all other voxels' time series (for *r* > 0.25).^8^ We obtained the unthresholded *t*-maps of voxel-wise degree centrality comparisons (found in Fig. 1(c) of Holiga *et al*^8^) for our analyses. To identify regions of significant hyperconnectivity (ASD > typically developed) and hypoconnectivity (typically developed > ASD), we performed a Gaussian random field cluster analysis on the unthresholded t-maps, using the FSL *cluster* tool^10^ distributed by the FMRIB Software Library (https://fsl.fmrib.ox.ac.uk/fsl/, v5.0, Mac). Smoothness parameters were estimated with FSL's *smoothest* utility and resulting values were input into the cluster utility, with an input threshold of *t* > 1.960 or *t* < −1.960 and a family-wise error (FWE) cluster-wise threshold of *P* < 0.05. The regions of hyperconnectivity and hypoconnectivity served as the inputs to behavioural profiling.

### Quantifying functional biases: meta-data collection

Meta-data were collected using the BrainMap Sleuth (v3.0.3, Mac) application.^6^ When an ROI is entered, the application returns foci of task-based fMRI studies that demonstrate activity (relative to a control task) within the ROI. The foci are all labelled with behavioural domain meta-data, allowing for subsequent analyses to determine how likely a behaviour is to activate the ROI. As Sleuth restricts ROIs to 1000 1 mm voxels, workspaces of ROIs that were greater than 1000 voxels were produced by entering the ROI as multiple smaller subregions to create an aggregate workspace encompassing all meta-data relevant to the original full ROI. For each input ROI, studies involving healthy participants and activations lying within the ROI were retained in the workspace. Hyperconnectivity clusters were input as one ROI, yielding a workspace for hyperconnectivity meta-data, and hypoconnectivity clusters were input as a second ROI, yielding a workspace for hypoconnectivity meta-data.

The ability to filter the workspace was added to the Sleuth application for the purpose of this study, allowing for the collection of experiments and foci to be filtered for particular behavioural domains. Experiments and foci for a particular ROI and behavioural domain were exported as formatted text files within the Sleuth application, with each text file being generated after filtering for a behavioural domain. Anderson and colleagues' multi-dimensional behavioural domain vector^4,11^ was modified to a 30-dimensional vector (largely based on Ref. 11) to include the following domains: anger, anxiety, attention, audition, disgust, execution, fear, gustation, happiness, imagination, inhibition, interoception, learning, mathematic, memory, music, observation, olfaction, orthography, phonology, preparation, reasoning, sadness, semantic, social, somesthesis, space, speech, syntax and vision. The 30 domains were chosen for their presence in BrainMap meta-data, their similarity to domains used in past work describing computational ability of brain regions, and the inclusion of domains relevant to ASD (i.e. the social domain). In addition to the 30 text files for hyperconnectivity ROIs and hypoconnectivity ROIs, 30 text files for a whole-brain ROI were generated to serve as the null hypothesis when performing inference on behavioural profiles.

The text files served as inputs to computationally rigorous meta-analyses. The amount of experimental data retained in text files could not be efficiently handled by a high-performance computing cluster during meta-analysis. So, a stratified random sample of 50% of the experiments was taken from the whole-brain ROI text files. Stratification allowed for a proportionate number of experiments per behavioural domain to be retained for meta-analysis. Experiments removed (i.e. not in the random 50% retained) from whole-brain ROI text files were removed from the hyperconnectivity and hypoconnectivity ROI text files. The amount to retain was set as 50% because previous meta-analyses using the high-performance computing cluster only succeeded when input with text files slightly under half the size (<200 kB) of the largest whole-brain ROI text file output by Sleuth. As the BrainMap database is in essence a sample of all possible task-based fMRI experiments, further sampling would not affect behavioural profiles. The resulting text files still contained data from many experiments, allowing for robust meta-analysis.

### Quantifying functional biases: construction of behavioural profiles

Behavioural profiles for each of the three hyperconnectivity ROIs and each of the two hypoconnectivity ROIs were created using the output of activation likelihood estimation (ALE).^12–14^ ALE finds the likelihood that an activation occurred at a brain coordinate given information on foci from experiments reporting activation near the coordinate.^15^ For each ROI, ALE values were used to determine the likelihood that a behavioural domain activated the ROI. ALE maps for each behavioural domain were produced for hyperconnectivity, hypoconnectivity and whole-brain ROI text files. BrainMap's GingerALE^12–14^ command-line tools (v3.0.2, Mac) were executed with Java (v1.8.0_181) on a high-performance computing cluster to write cluster-level FWE-corrected ALE maps. The cluster-level FWE method is considered to be the most appropriate for meta-analysis inference.^16^ The parameters used for the FWE- corrected ALE were a *P*-value threshold of 0.001, a secondary cluster-forming threshold of 0.05 and 1000 permutations. As Sleuth retains experiments with foci within ROIs but also retains foci outside the ROIs (which are coactivation partners to within-ROI foci), ALE maps from the hyperconnectivity text files were masked by the three hyperconnectivity ROIs and ALE maps from the hypoconnectivity text files were masked by the two hypoconnectivity ROIs. The masking generated ALE maps that described the likelihood that a behavioural domain would activate voxels in a given ROI (where ROIs are the five clusters of hyper/hypoconnectivity).

**Fig. 2:**
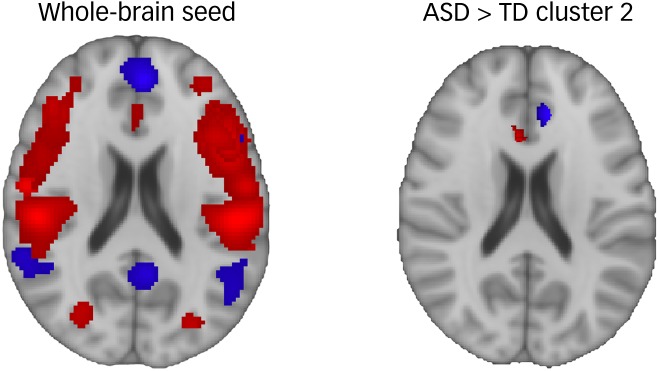
Behavioural profiling of ALE maps.

Behavioural profiles were calculated directly from masked ALE maps using SPM (SPM12) in MATLAB (v2019a, Mac). Following from published methods,^4^ an active voxel served as an observation, and the weight of the observation was determined by the ALE value. ALE values were summed from each ALE map to determine one value per ROI per behavioural domain that represented the magnitude of the region's involvement in the given behavioural domain (Fig. 2). The 30 magnitudes for a region's behavioural domains were normalised to represent the proportions by which the behavioural domains were involved in the brain region. The behavioural profiles were corrected for the zero-occurrence issue using Anderson's^4^ smoothing methods based on the work of Jelinek and Mercer.^17^ Summed ALE maps equal to zero could inaccurately suggest that a brain region had zero involvement in a certain task domain. Thus, following from published work,^4^ all behavioural profile proportions for regions were smoothened based on the whole-brain behavioural profiles, in which the whole brain acted as a seed region for the Sleuth application. Calculations of smoothened proportions for behavioural profiles used the following equation:
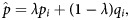
where 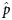 is the smoothened proportion, *p*_*i*_ is the regional proportion for domain *i* and *q*_*i*_ is the whole-brain proportion for domain *i*. *λ* is a sigmoidal function that allowed the proportions to be adjusted according to how many active voxels, *n*, were observed from the domain's GingerALE map. The sigmoidal curve of Anderson^4^ was used, as its exponent scaling also fit the data for this procedure. The function was defined as:
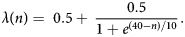

ALE maps for each of 30 behavioural domains are overlaid for the whole-brain maps and hyperconnectivity cluster 2 maps, with the blue ALE maps corresponding to the ‘social’ behavioural domain, and for all other behavioural domain ALE maps shown in red. The proportion of behavioural domain representation is computed based on the sum of all voxels in the ALE map, and the spatial extent of the ALE maps in the images can be used to roughly visualise the proportion. Hyperconnectivity (ASD > TD) cluster 2 is used here as an example to depict the relationship between ALE maps and behavioural profile proportions. It can be seen that the social ALE map is represented in large proportion compared with the other ALE maps in hyperconnectivity cluster 2 (right image) and that the social ALE map is in higher proportion in hyperconnectivity cluster 2 than the social ALE map in the whole-brain seed (left image). The large proportion compared with within-cluster ALE maps demonstrates that social behaviour is dominantly represented in hyperconnectivity cluster 2, and the higher proportion compared to the social ALE map in the whole-brain seed depicts the significant overrepresentation of social behaviour in hyperconnectivity cluster 2. ALE, activation likelihood estimation; ASD, autism spectrum disorder; TD, typically developed.

An increased number of active voxels led to behavioural domain magnitudes being based more on the region's proportion, and a decreased number of active voxels led to behavioural magnitudes being based more on the whole brain's proportion.

### Analysis of behavioural profiles

Behavioural profiles were analysed to determine whether a behaviour was dominantly represented in the region and was more represented in the region compared to the whole brain. Behavioural domains with the largest proportions and whose proportions added to at least 60% were considered to be dominantly represented in the brain region. The value of 60% was chosen as it did not exclude any behavioural domains with large proportions (< 10%) and would account for most of the behavioural representation. A single-tailed one-proportion *z*-test was used to determine whether increases in proportions in regions were significant compared to the whole-brain null-profile. The z-score calculation used the equation:
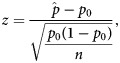
where 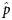 is the normalised proportion for a given ROI and behavioural domain, *p*_0_ is the proportion for which the null distribution is involved in a behavioural domain and *n* is the total number of voxels with ALE values greater than zero from all the ALE maps for a given ROI. *P*-values were Bonferroni corrected as there were significance tests for many behavioural domains.

## Results

### Clusters of atypical functional connectivity

**Fig. 3:**
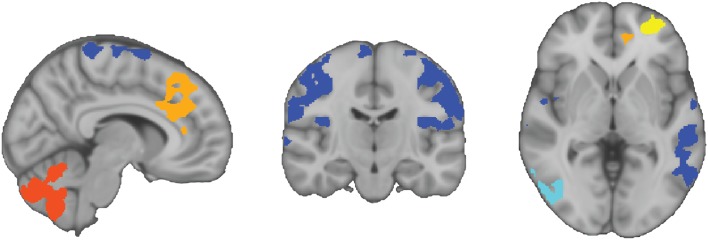
Autism spectrum disorder hyperconnectivity and hypoconnectivity clusters.

Five clusters of atypical connectivity were output from the FWE-corrected cluster analysis (Fig. 3). Three clusters of hyperconnectivity were observed. Cluster 1 extended across the middle frontal gyrus and frontal pole. Cluster 2 extended across the anterior cingulate gyrus, paracingulate gyrus and superior frontal gyrus. Cluster 3 was contained within the cerebellum. Two clusters of hypoconnectivity were observed. Cluster 1 extended across the inferior temporal gyrus and lateral occipital cortex. Cluster 2 extended across the postcentral and precentral gyrus, central opercular cortex, parietal operculum cortex, lateral occipital and middle temporal gyrus.

Hyperconnected clusters are highlighted in warm colours (yellow = cluster 1, orange = cluster 2, red = cluster 3), and hypoconnected regions are highlighted in cool colours (light blue = cluster 1, dark blue = cluster 2). All hyper/hypoconnectivity overlays are in binary format, and the colours are only used to distinguish the clusters. Sagittal, coronal and axial slices in the image correspond to *x* = 8, *y* = −17 and *z* = −1 in MNI152 coordinates.

### Behavioural profiles

**Fig. 4:**
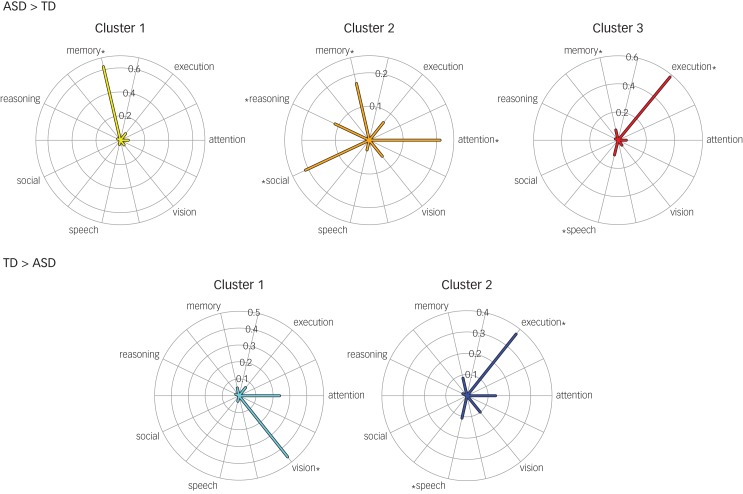
Behavioural profiles for each hyperconnectivity and hypoconnectivity cluster.

Hyperconnected and hypoconnected brain regions had functional biases toward various behavioural domains (Fig. 4). Although the data from each of 30 behavioural domains were used to compute behavioural profiles and evaluate significance for each behavioural domain, only the behavioural domains that reached significance in any of the five behavioural profiles constructed are shown in Fig. 4 to highlight variation across clusters in the representation of these most relevant behavioural domains. Hyperconnected regions in ASD brains had functional biases towards memory, attention, reasoning, social, execution and speech behavioural domains. Hypoconnected regions in ASD brains had functional biases towards vision, execution and speech behavioural domains.

Behavioural domains that reached significance in any of the five clusters are listed on each of the radial plot's axes, and the value along the axis through which the line segments extend indicates the proportion of the brain region's involvement in the behavioural domain. The behavioural profiles visualise the overrepresented behavioural domains in each cluster. Asterisks next to behavioural domains denote significant functional biases within the cluster (*P* < 0.0001). TD, typically developed; ASD, autism spectrum disorder.

All significant functional biases were significant at *P* < 0.0001. Memory was a significant functional bias for hyperconnected cluster 1. It should be noted the zero-occurrence issue was encountered for all domains except memory in this region. Attention, memory, reasoning and social were all significant functional biases for hyperconnected cluster 2. Execution and speech were both significant functional biases for hyperconnected cluster 3. Vision was a significant functional bias for hypoconnected cluster 1. Execution and speech were both significant functional biases for hypoconnected cluster 2.

## Discussion

We first identified regions of atypical connectivity in ASD brains from a previously published resting state functional connectivity comparison. We applied a novel behavioural profiling method to the regions showing atypical connectivity to characterise the degree to which these regions were implicated in behavioural domains associated with ASD. The behavioural profiles of brain regions with altered connectivity suggest that ASD brain hyperconnectivity may contribute to memory, attention, reasoning, social, execution and speech deficits, whereas hypoconnectivity may contribute to vision, execution and speech behavioural deficits. This behavioural profiling method will also be valuable for investigating a variety of behavioural domains in neurodevelopmental and psychiatric disorders outside ASD.

Various quantitative reverse-inference methods have been applied to understand disease and disorder, and behavioural profiling represents one method designed in particular to investigate the heterogenous pathology in ASD. Müller *et al*^18^ described one of the first applications of meta-analysis to infer the function of brain regions affected by depression; more recently, behavioural time series were constructed to investigate brain disruptions in ASD.^19^ Other methods exist, but we distinguish behavioural profiling from other forms of quantitative reverse-inference in how it represents the diversity of function for a given region, rather than the behavioural domains that most significantly activate a region. This is reflected in how the computed proportion of a behavioural domain's involvement in a behavioural profile is dependent on the proportions of other behavioural domains. Thus, the mappings of behaviour to brain regions in a behavioural profile reveal functional biases, and the range of functional biases speak to the functional capacity of underlying neural computations. We chose to use hyperconnectivity and hypoconnectivity differences to account for the relationships among connectivity, regional computations and behaviour. A combination of hyperconnectivity and hypoconnectivity has been a repeated finding in the ASD literature.^20^ The present work sought to disentangle how brain regions contribute to pathology. Connectivity abnormalities can sometimes be associated with certain cognitive effects, such as hyperconnectivity being associated with hypersensitivity^21^ and the relative decreased connectivity implied by hypoconnectivity being associated with relative decreased social cognitive ability.^22^ Instead, we found visual/sensory functional bias in hypoconnected regions and social functional bias in hyperconnected regions. The results indicate a potential role for hyperconnectivity in driving ASD repetitive behaviours (execution), social cognitive deficits (social), language deficits (speech) and other cognitive deficits (memory, attention, reasoning); and a role for hypoconnectivity in driving ASD repetitive behaviours (execution) and sensory hypersensitivity (vision). The manner in which underlying brain computations are affected may not be entailed by the directionality of the connectivity deficit (hyper versus hypo), which emphasises the utility of quantitative reverse inference methods such as behavioural profiling in potentially dissociating features of connectopathies.

Anderson *et al*'s^4^ findings of brain region functional diversity additionally challenged computational compartmentalisation theories of functional brain architecture, which are increasingly falling out of vogue. A computational framework based on functional diversity of brain regions may be useful for understanding a wide variety of neurodevelopmental disorders. In this framework, underlying brain computations are functionally biased by the brain region in which they are located, but the computations are flexible in that they can be recruited for various tasks. Computational reuse accounts for functional diversity and processing biases, as computations can be recruited for tasks in a variety of domains while also exhibiting domain-preferences (i.e. biases) that reflect the tasks that the brain regions are ‘best at computing’. Anderson^23^ (p. 59) has described learning as a process by which the brain finds which brain regions are best at computing certain tasks; these brain regions are more likely to be recruited for those tasks and task-based networks because of their functional biases. Functional biases demonstrate how computationally biased a brain region is for a certain behavioural domain, and tasks are therefore more often carried out via brain regions suited to the task's behavioural domain.

The computational reuse framework has important implications for features of neurodevelopmental disorders, and the behavioural profiling approach provides novel insights into computational reuse. The multicausality, multifinality and transdiagnostic features of neurodevelopmental disorders can be accounted for in the framework. Multicausality reflects the convergence of causal factors on common functional features of neurons or neuron populations, thereby creating computation-specific functional abnormalities. Multifinality, or behavioural heterogeneity, as seen in ASD, could reflect individual differences in functional biases of affected brain regions or could reflect computational comorbidity, in which a single causal factor affects core computations of behaviours affected in ASD, while also affecting a variety of other computations. Overlapping etiology^24–26^ and brain abnormalities^27–30^ across neurodevelopmental disorders may reflect common computational deficits that are evident in common behavioural deficits. The computational context of functional brain abnormalities will be valuable for describing these features of neurodevelopmental disorders, and the behavioural profiling method described here allows fMRI experimental paradigms to give computational context to determined functional abnormalities. The method can quantify the degree to which the determined affected brain regions are computationally relevant to behaviours affected by the disorder. Other methods have incorporated task-based neuroimaging data to either establish ROIs *a priori*^31^ or to determine behaviours most related to affected ROIs;^32^ however, the behavioural profiling method paradigmatically differs from the aforementioned methods in how it treats ROIs. An ROI is treated as a ‘black box’ that can only be analysed by its functions, and the behavioural profile of the ROI is not merely a probabilistic inference of the ROI's connection to behaviour but rather a holistic description of the black box's intrinsic functional capacities. For any ROI, healthy subject data can be used to describe the brain-to-behaviour relationship in terms of a behavioural profile. Applying a computational reuse framework, the ROI performs some unknown (i.e. black box) neural computation that is most relevant to certain behaviours. If this brain region is affected in a disorder, the neural computation is also likely to be affected. Therefore, by characterising ‘unhealthy’ ROIs with healthy participant data, we are indirectly characterising the healthy neural computations that are disrupted in a disorder. From there, it would be coherent that the neural computations, through behavioural profiling, suggest an important role for behaviours known to be affected by the disorder.

This behavioural profiling method uses meta-analytic data derived from a sample of typical/healthy brains. One might argue that brain region-to-behaviour assumptions may not be valid in patients with brain disorders and therefore this step should focus on using meta-analytic data from disordered brains. However, using ROIs based on meta-data from healthy brains actually allows for the characterisation of the cognitive functions that the disrupted regions should be engaged in. That is, if we assume that atypical behaviours and cognitive functions in developmental disorders are due to abnormal use of a given brain region, it follows that abnormalities will occur in regions typically associated with behaviours affected by the disorder. By finding which regions with functional abnormalities are most relevant to the disorder in this manner, the connections among the biological, computational and functional (as seen at the fMRI level) manifestations of causal factors can be made clearer. Thus, this behavioural profiling method may be especially useful when the condition studied is heterogeneous across patients. Shared computational disruptions can be reflected in behavioural profiles of shared connectivity disruptions.

### Limitations

Although there may be biases in the type of meta-data reported to BrainMap, we consider the database to provide a large enough sample for behavioural domain meta-data to enable comparisons of the degree of meta-data related to certain brain regions to be validly compared to that of the whole brain. In addition, the 30 behavioural domains used for the brain region descriptions were chosen to include as much meta-data as possible without domain overlap, again with the intent of allowing valid statistical comparison of brain region sample meta-data with whole-brain sample meta-data. The multidimensional behavioural domain vector was adapted from the published work of Anderson and colleagues,^4^ who also employed statistical comparisons between behavioural profiles. Healthy participant meta-data with no age restriction were used because of the ubiquity of the data, especially in comparison with the relatively sparse ASD meta-data within the database. We assumed that the gross localisation of functional brain architecture was comparable between typically developed individuals and individuals with ASD and among individuals of different ages when employing the behavioural profiling method, but it may be interesting to investigate the developmental trajectories of brain regions' behavioural profiles in future work.

There were a considerable number of zero-occurrence events in constructing the ROIs' behavioural profiles. However, there was only one behavioural domain in the hyperconnected text files (fed into ALE) and one in the hypoconnected text files for which there were no experiments listed. Therefore, most of the zero-occurrence cases were not because of a lack of data but because there were zero positive ALE values in the behavioural domain ALE maps; this was because either the cluster-level FWE ALE did not find any significant clusters, or the significant clusters were outside the ROI being investigated (e.g. hyperconnected cluster 1). The smoothing procedure makes estimates of functional bias more conservative, adding to the validity of the functional bias estimates when zero-occurrence cases were present.

### Future directions

Methods including regional homogeneity, between-ness centrality of nodes, seed-based analysis, independent component analysis (ICA)-dual regression, resting network and task network correlation and graph theory have been applied to understand ASD brain function, all giving different perspectives on the neural manifestation of ASD.^21^ As each method has varying implications for what drives group-level differences in the brain, behavioural profiling of functional differences as revealed by different methods can further connect pathology to behaviour. Future work may extend the frameworks of behavioural profiling and computational reuse to the individual level. Anderson^33^ suggested analyses of individual person's brain region profiles over time as a way to investigate developmental trajectories, for example. Innovative methods that take advantage of meta-analytic databases will be useful for the continued characterisation of ASD and other conditions at the group and individual levels.
