# Behavioral profiling of autism connectivity abnormalities – Erratum

**DOI:** 10.1192/bjo.2020.19

**Published:** 2020-04-07

**Authors:** William Snyder, Vanessa Troiani

**Keywords:** Autism spectrum disorder, neuroimaging, fMRI, meta-analysis

Author William Snyder's affiliation erroneously included PhD in his biography. The correct biography is:

**William Snyder**, Student, Program in Neuroscience, Bucknell University; Research Assistant, Autism and Developmental Medicine Institute, Geisinger, USA

This article was incorrectly published with an extra asterisk in Figure 4. The correct figure is below.

**Fig. 4:**
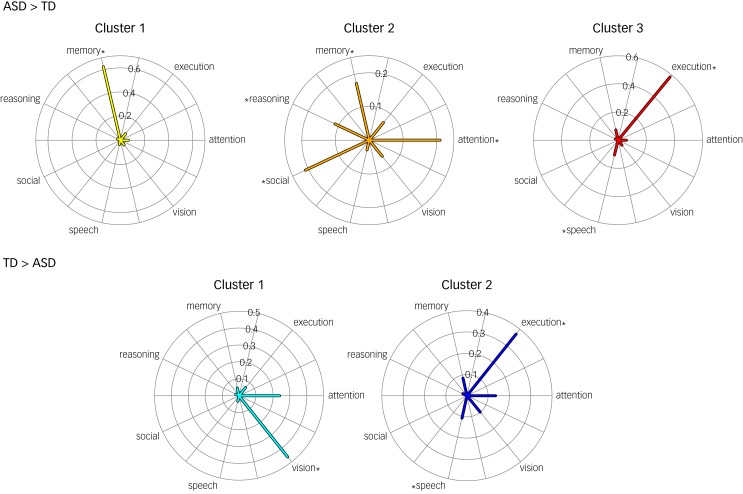

The publisher sincerely apologises for this error.
